# Analysis and Compensation of Phase Current Measuring Error Caused by Sensing Resistor in PMSM Application

**DOI:** 10.3390/s24051538

**Published:** 2024-02-28

**Authors:** Xin Cheng, Jinfeng Hu, Ye Yu, Rougang Zhou, Qiang Yu

**Affiliations:** 1 School of Information Engineering, Wuhan University of Technology, Wuhan 430070, China; 2 School of Mechanical & Electronic Engineering, Wuhan University of Technology, Wuhan 430070, China; 318608@whut.edu.cn (J.H.); yuyewy121@163.com (Y.Y.); yuqiang_lys@163.com (Q.Y.); 3 School of Mechanical Engineering, Hangzhou Dianzi University, Hangzhou 310000, China; 4 Wenzhou Institute of Hangzhou Dianzi University, Wenzhou 325013, China; 5 Mstar Technologies, Inc., Hangzhou 310012, China

**Keywords:** permanent magnet synchronous motor, field oriented control, current sampling, error analysis and compensation, PI observer

## Abstract

Field Oriented Control (FOC) effectively realizes independent control of flux linkage and torque, and is widely used in application of Permanent Magnet Synchronous Motor (PMSM). However, it is necessary to detect the phase current information of the motor to realize the current closed-loop control. The phase current detection method based on a sampling resistor will cause a measurement error due to the influence of parasitic parameters of the sampling resistor, which will lead to the decrease in PMSM control performance. This paper reveals the formation mechanism of the current sampling error caused by parasitic inductance and capacitance of the sampling resistor, and further confirms that the above error will lead to the fluctuation of the electromagnetic torque output by simulation. Moreover, we propose an approach for online observation and compensation of the current sampling error based on PI-type observer to suppresses the torque pulsation of PMSM. The phase current sampling error is estimated by the proportional and integral (PI) observer, and the deviation value of current sampling is obtained by low-pass filter (LPF). The above deviation value is further injected into the phase current close-loop for error compensation. The PI observer continues to work to keep the current sampling error close to zero. The simulation platform of Matlab/Simulink (Version: R2021b) is established to verify the effectiveness of online error observation and compensation. Further experiments also prove that the proposed method can effectively improve the torque fluctuation of the PMSM and enhance its control accuracy performance of rotation speed.

## 1. Introduction

PMSM is increasingly applied to electric vehicles, robots, and marine propulsion due to high power density, wide speed range, strong anti-interference capability, and high efficiency [1,2,3,4,5]. Considering the rotor structure, motor size, and security in industrial application, the installation of speed/position sensor is not recommended, and consequently, sensorless control methods of PMSM have emerged [6,7,8]. FOC realizes independent control of the flux linkage and torque by utilizing the Clark and Park transformations, and results in enhancements of PMSM’s performance [9,10].

FOC needs phase currents information as feedback signals to realize the closed-loop control of the current [11]. The current loop is the innermost loop of FOC; its performance constrains the performance of the entire control system [12,13]. The current sampling is a crucial step in acquiring phase current information, and the current sampling errors will cause torque pulsation of PMSM and affect the control performance of FOC [14,15].

Sources of current measurement errors can be categorized as follows. (1) Gain error is caused by the unequal proportional gain of the current sensors, operational amplifiers, and the passive components in conditioning circuits, which may change with time and temperature [16,17,18]. The presence of gain error will generate the torque ripple, leading to the mechanical vibration and decreasing service life [19,20]. (2) Offset error [21,22] is a superimposed quantity, which is caused by unstable power supply voltage, the deadband of pulse wide modulation (PWM), and offset error in sensors. Offset errors generate harmonic components in the torque with the same frequency as the stator current, which will provide wrong amplitude information to the deadband compensator in the power stage [23].

For the current measurement error, many scholars carry out in-depth discussions and propose many compensation schemes. Bai Y, Li B, Wang Q, et al. [24] constructed a single frequency current harmonic suppressor to filter out the harmonic currents caused by the current sensor measurement error from the current harmonic suppression consideration, which is feasible at the low-speed operation of PMSM, but lacks the suppression effect at the high-speed operation of PMSM. Lei M, Peng T, Zhou F, et al. [25] proposed an improved temperature compensation circuit to suppress the temperature drift of the current sensor, which fundamentally solves the phase current offset errors due to temperature shift, but neglects the current offset errors caused by the supply voltage unbalance problem of the current sensor. Yamaguchi T, Tadano Y and Hoshi N [26] proposed a periodic disturbance observer to suppress harmonic current, considering that the current offset error caused by the current sensor will cause harmonic currents at specific frequencies in the q-axis, and the observer will suppress the current signals at those specific frequencies, which can compensate the gain error offset error at the same time. Zhang K, Fan M, Yang Y, et al. [27] proposed an improved harmonic elimination algorithm to eliminate the q-axis harmonic current caused by the current measurement errors, which effectively suppresses the harmonic currents in the q-axis, but increases the difficulty of phase current reconstruction. Zhang Q, Guo H, Liu Y, et al. [28] proposed an error compensation strategy of human error injection to reduce the offset error and gain error by multiple human error compensation injections, which effectively compensates the offset error and gain error of the phase current, but the experimental process is more cumbersome. Ye S and Yao X [29] proposed an improved sliding mode observer that integrates the current error compensator into the sliding mode observer to compensate the offset error and gain current error in the current error measurement, which can effectively compensate the current measurement error, but the shake of the motor will affect the performance of the observer. Kim S I, Kim J Y and Lee K W [30] proposed a current offset error compensator, which reflects the error between the output of the current controller and the actual output voltage to the current measurement offset error compensator to compensate the current offset error.

In the PMSM control systems that use sampling resistors as current sensors, the effect of sampling resistor parasitic parameters on current sampling is often neglected. In this paper, the current offset error caused by sampling resistor parasitic inductance in the current sampling process is investigated, and an error compensation method is pre-proposed to compensate the current offset error. 

The main contributions of the paper are highlighted as follows.

- We reveal the effect of parasitic capacitance and inductance of the sampling resistor on the electromagnetic torque characteristics of PMSM.
- We propose an online observation and compensation method of current sampling error based on the PI-type observer, which effectively suppresses the torque pulsation of PMSM.
- The parameters of the PMSM are achieved from the laboratory.

The rest of this paper is organized as follows. The model of the current measurement error is presented in Section 2. We introduce an online observation and compensation method of current sampling error based on the PI-type observer in Section 3. The experimental platform and results are discussed in Section 4. Section 5 gives the conclusions and future work. 

## 2. Current Measurement Error Model

### 2.1. Current Sensors in PMSM

The current sensor plays a crucial role in the PMSM drive control system; it feeds the sampled phase current information back to the current loop, and the accuracy of phase current sampling directly affects the stability of the PMSM drive control system [31,32]. During long term operation, the performance of PMSM can be affected by various environmental factors such as vibration impact, high and low temperature, humidity, and dust. These factors can cause current sensor sampling failure and impact the stability of PMSM operation [33,34]. To address the drawbacks of using current sensors, a sampling resistor is often connected in series between the lower arm of the inverter and the ground in engineering, which acts as the current sensor [35]. The sampling resistor is a low resistance resistor with high precision and low temperature drift, and has good application stability and reliability.

**Figure 1:**
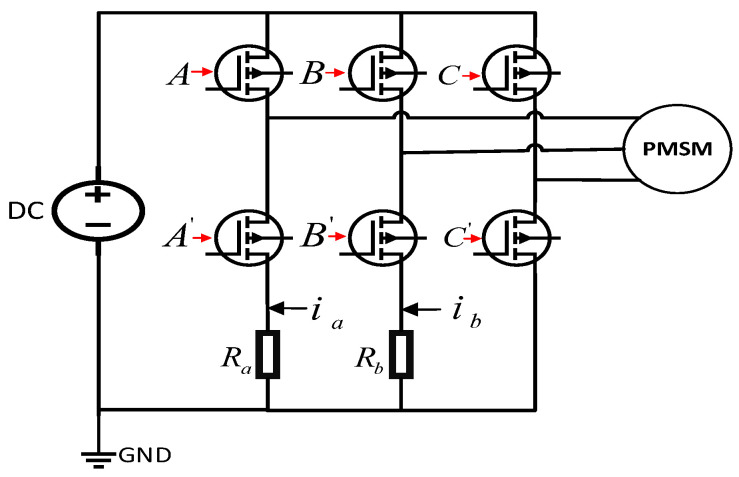
Three-phase inverter topology with dual sampling resistors.

According to the different channels of current sampling, the sampling methods can be divided into three types [36,37,38]: three-phase current sampling, two-phase current sampling, and single-phase current sampling. In this paper, the three-phase inverter topology with two-phase current sampling constructed for the sampling resistor is shown in Figure 1.

### 2.2. Sources of Current Sampling Errors

The sampling resistor is affected by its material and manufacturing process; the sampling resistor will have parasitic parameters, such as parasitic capacitance and parasitic inductance [39]. Considering the existence of parasitic parameters, the sampling resistor behaves as series and parallel model of resistance, capacitance, and inductance in the actual circuit [40]. Using the impedance to describe the blocking effect of the sampling resistor on the current, and the impedance magnitude can be described as:

(1) $$Z=R+j(\omega L-\frac{1}{\omega C})$$

where: *Z*, *R*, *L*, and *C* are impedance, actual resistance, parasitic inductance, and capacitance of sampling resistor, respectively. *ω* is the frequency of phase current.

For the PMSM drive control system that uses sampling resistors, the current is sampled by the analog-to-digital converter (ADC) of the control chip after passing through the operational amplifier and other conditioning circuits. The current value can be reflected by the value of the potential difference on the sampling resistor [41]. Phase current values sampling by ADC can be expressed as:

(2) $$\left\{ \begin{array}{l} i_{a\_AD}=Z_{a}\cdot i_{a\_\mathrm{act}}\cdot K_{opa\_a}\\ i_{b\_AD}=Z_{b}\cdot i_{b\_\mathrm{act}}\cdot K_{opa\_b} \end{array}\right.$$

where: *i_a_AD_* and *i_b_AD_* are the sampling value of ADC corresponding to the phase current, respectively; *Z_a_* and *Z_b_* are the value of impedance corresponding to the a-phase and b-phase sampling resistor, respectively; *i_a_act_* and *i_b_act_* are the actual value of the phase current, respectively; *K_opa_a_* and *K_opa_b_* are the gain of amplifier circuits of the a-phase and b-phase, respectively.

Considering the frequency of the phase current and the value of the parasitic capacitance of the sampling resistor, the influence of the parasitic capacitance of the sampling resistor on the impedance can be ignored. The high precision resistor with 1% or 0.5% accuracy is commonly used as the sampling resistor in engineering; thus, the impact of the actual value and the theoretical value of the sampling resistance can be ignored. According to Equations (1) and (2), phase current values sampling by ADC of the chip can be presented in the following form:

(3) $$\left\{ \begin{array}{l} i_{a\_AD}=R_{a}\cdot i_{a\_\mathrm{act}}\cdot K_{opa\_a}+j\omega L_{a}\cdot i_{a\_\mathrm{act}}\cdot K_{opa\_a}\\ i_{b\_AD}=R_{b}\cdot i_{b\_\mathrm{act}}\cdot K_{opa\_b}+j\omega L_{b}\cdot i_{b\_\mathrm{act}}\cdot K_{opa\_b}\\ i_{a\_AD}=i_{a}+i_{a\_offset}\\ i_{b\_AD}=i_{b}+i_{b\_offset} \end{array}\right.$$

where: *R_a_* and *R_b_* are theoretical values of the sampling resistors of a-phase and b-phase, respectively; *i_a_* and *i_b_* are theoretical measured values of a-phase and b-phase currents, respectively. Additionally, *i_a_offset_* and *i_b_offset_* are offset currents, which are caused by the parasitic inductance of the sampling resistor during the measurement of phase currents. After considering the impact of above factors, the theoretical measured values and theoretical offset values of phase current can be expressed in the following form:

(4) $$\left\{ \begin{array}{l} i_{a}=R_{a}\cdot i_{a\_\mathrm{act}}\cdot K_{opa\_a}\\ i_{b}=R_{b}\cdot i_{b\_\mathrm{act}}\cdot K_{opa\_b}\\ i_{a\_offset}=j\omega L_{a}\cdot i_{a\_\mathrm{act}}\cdot K_{opa\_a}\\ i_{b\_offset}=j\omega L_{b}\cdot i_{b\_\mathrm{act}}\cdot K_{opa\_b} \end{array}\right.$$

The sampling resistor resistance error and sampling resistor parasitic capacitance error have been neglected. According to the expression of the theoretical measurement value and the theoretical offset value of the phase current, the phase current measurement error mainly comes from the phase current offset error, which is caused by the parasitic inductance of the sampling resistor.

### 2.3. Effects of Current Sampling Error

**Figure 2:**
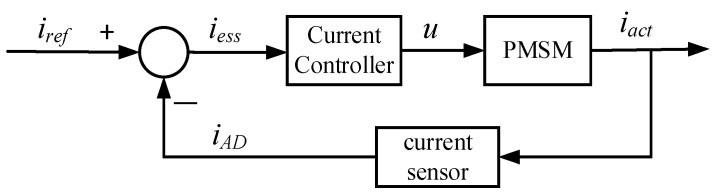
Control block diagrams of the current loop.

The speed regulation of the PMSM is realized by the closed-loop control of the current and speed, and the three-phase inverter regulates the PMSM’s speed based on the current error [42]. Figure 2 shows the block diagram of the current loop, *i_ref_*, *i_ess_*, *i_act_*, and *i_AD_* are the reference current, current error, actual current, and the sampling current of the control chip’s ADC, respectively. *u* is the command voltage.

Ideally, the current loop error is 0, but the reference current value and the current sampling value are not equal in actual operation. There is an error in the feedback of actual current, but it cannot be reflected directly from the current sampling value, so it is necessary to find other variables that reflect the current error.

In the closed-loop control system of FOC, the coordinate transformation of the sampling current is performed. After the Clark transformation, the current offset error in Equation (4) is expressed in α-β coordinate system as:

(5) $$\left\{ \begin{array}{l} i_{\alpha \_m}=i_{\alpha }+i_{\alpha \_offset}\\ i_{\beta \_m}=i_{\beta }+i_{\beta \_offset}\\ i_{\alpha \_offset}=i_{a\_offset}\\ i_{\beta \_offset}=\frac{\sqrt{3}}{3}(i_{a\_offset}+2i_{b\_offset}) \end{array}\right.$$

where: *i_α___m_* and *i_β___m_* are measured currents of α-axis and β-axis, respectively; *i_α___offset_* and *i_β___offset_* are current offset errors of α-axis and β-axis, respectively.

After Park transformation of the current offset error in the α-β coordinate system, the expression of the current offset error in the d-q coordinate system can be presented in the following form:

(6) $$\left\{ \begin{array}{l} i_{d\_m}=i_{d}+i_{d\_offset}\\ i_{q\_m}=i_{q}+i_{q\_offset}\\ i_{d\_offset}=K_{offset}sin(\omega_{r}+\phi )\\ i_{q\_offset}=K_{offset}cos(\omega_{r}+\phi ) \end{array}\right.$$

where: *i_d_m_* and *i_q_m_* are measured currents of q-axis and d-axis, respectively; *i_d_offset_* and *i_q_offset_* are current offset errors of q-axis and d-axis, respectively; *K_offset_*, *ω_r_* and ϕ are the amplitude, frequency and phase of the current offset error, respectively.

The expressions in Equation (6) can be transformed to obtain expressions *K_offset_* and ϕ, which can be expressed as:

(7) $$\left\{ \begin{array}{l} K_{offset}=\sqrt{i_{a\_offset}^{2}+\frac{1}{3}{\left(i_{a\_offset}+2i_{b\_offset}\right)}^{2}}\\ \phi =\arctan[2(\sqrt{3}i_{a\_offset},i_{a\_offset}+2i_{b\_offset})] \end{array}\right.$$

**Figure 3:**
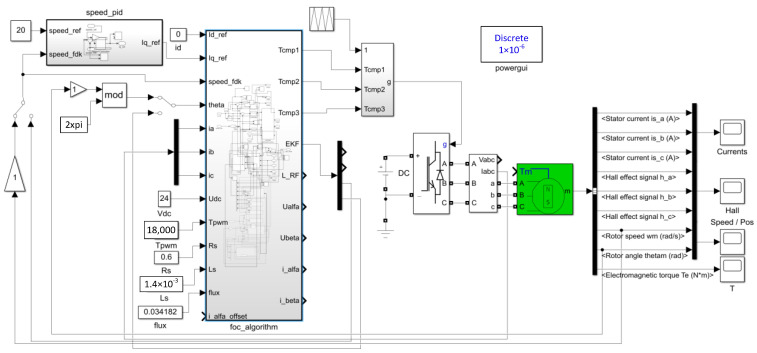
Simulation model of FOC based PMSM drive control system.

The simulation model of FOC based PMSM drive control system is built in Matlab/Simulink. The simulation sampling step time is 1 × 10^−6^ s. The motor model is a surface-mounted PMSM from the Simulink library. The initial load is 0 N·m, and the rotational speed frequency of motor is 20 Hz. The PI controller is selected from the Simulink library and the anti-integral saturation is turned on, and the q-axis current amplitude is limited to 25 A. PI parameters of speed loop are *K_p_* = 0.0025 and *K_i_* = 4.8, while PI parameters of current loop are *K_p_* = 2.15 and *K_i_* = 1281.5. The power of the motor is small, and Metal Oxide Semiconductor (MOS) tubes are chosen to form the three-phase full-bridge circuit of the inverter. Simulation model of FOC based PMSM drive control system is shown in Figure 3.

**Figure 4:**
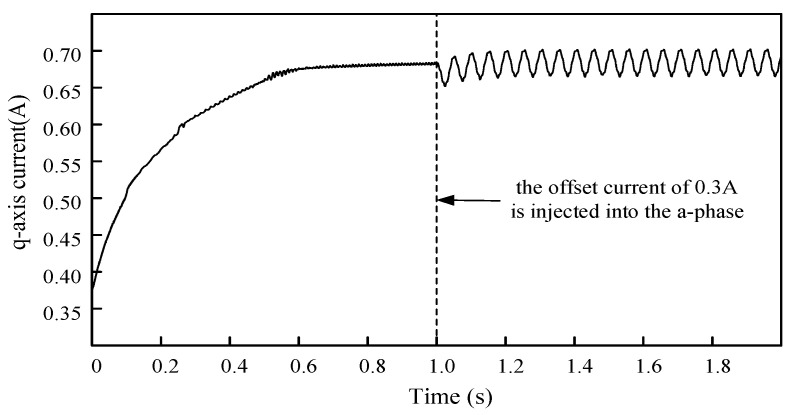
The effect of the injected offset current into a-phase on the q-axis current of the PMSM. An offset current of 0.3 A was injected into a-phase at 1 s.

The frequency of reference speed is 20 Hz in the PMSM drive control system, and the offset current of 0.3 A is injected into the a-phase at 1 s. Waveform of the q-axis current obtained by simulation in Simulink, as shown in Figure 4.

**Figure 5:**
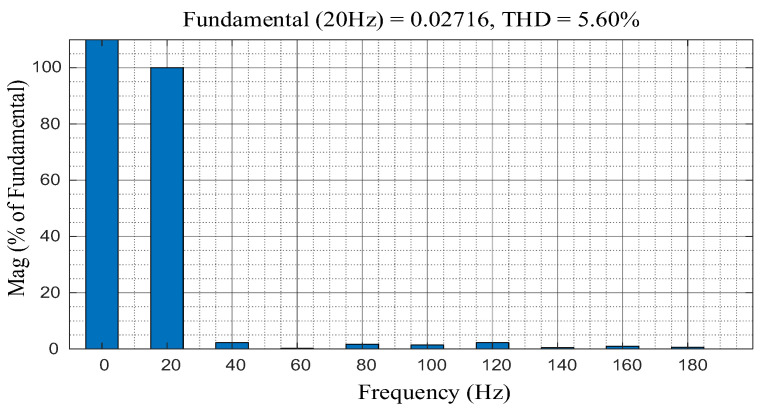
The FFT result of q-axis current of the PMSM in Figure 4.

The q-axis current in Figure 4 is analyzed using fast Fourier transformation (FFT) to determine the waveform distortion rate and frequency of the oscillation component. The FFT result is shown in Figure 5.

As shown in Figure 5, the total harmonic distortion (THD) of q-axis current is 5.60%, it indicates that the frequency of the alternating current (AC) oscillation component generated on the q-axis current is close to 20 Hz, and confirms the derivation of Equation (6). It also confirms that the current offset error appears as an AC oscillation in q-d axis, and the oscillation frequency is close to the operating frequency of PMSM.

The motor model in Simulink is the surface-mounted PMSM, flux distribution of it is uniform, so the q-axis and d-axis inductances are equal [43]. Equation (6) is brought into the torque equation of the PMSM [44], which is reconstructed using measured q-axis current and offset current to obtain a new torque equation, and the new torque equation can be expressed as:

(8) $$\left\{ \begin{array}{l} T_{e}=\frac{3}{2}P_{n}\Psi_{f}(i_{q\_m}-i_{q\_offset})\\ T_{e}=T_{e\_ref}-\Delta T\\ T_{e\_ref}=\frac{3}{2}P_{n}\Psi_{f}i_{q\_m}\\ \Delta T=\frac{3}{2}P_{n}\Psi_{f}i_{q\_offset} \end{array}\right.$$

where: *P_n_*, *Ψ_f_*, and *T_e_ref_* are the pole pair number, the permanent magnet flux linkage, and the commanded torque of PMSM, respectively; ∆*T* is the torque fluctuation caused by the offset current error.

**Figure 6:**
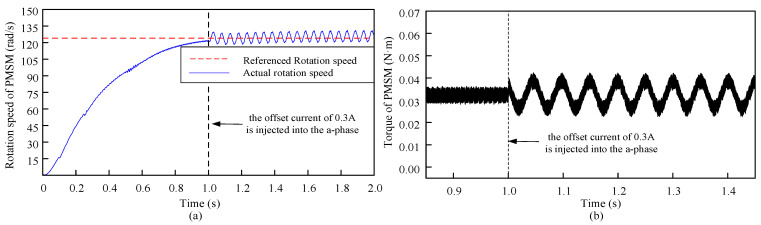
The effect of the injected offset current to a-phase on the rotation speed and the torque of the PMSM. An offset current of 0.3 A was injected into a-phase at 1 s. (**a**) The rotation speed waveform of PMSM (The red dotted line and blue are referenced rotation speed and actual rotation speed, respectively); (**b**) The torque waveform of PMSM.

The rotation speed and torque waveform of PMSM can be obtained while obtaining the q-axis current waveform in Simulink, which is shown in Figure 6.

From Figure 6, the offset current error will cause speed and torque fluctuations in FOC, and the generated torque ripple will not only lead to increased loss and decreased average torque of PMSM during PMSM operation, but also cause mechanical vibration.

## 3. Online Observation and Compensation Methods for Current Measurement Errors

### 3.1. Overall Design

The sources of current offset error and the error influence on PMSM drive control system are analyzed, how to eliminate the current offset error and reduce the torque ripple of the PMSM becomes the key problem to be solved. In the PMSM drive control system, observers such as Luenberger observer, sliding mode observer, and Kalman filter observer, are commonly used to observe the angle or speed information of PMSM. Based on the above consideration, the observer is designed to observe the current offset error, analyze its impact in open-loop control, and build the simulation model in Simulink to verify the observer’s performance.

**Figure 7:**
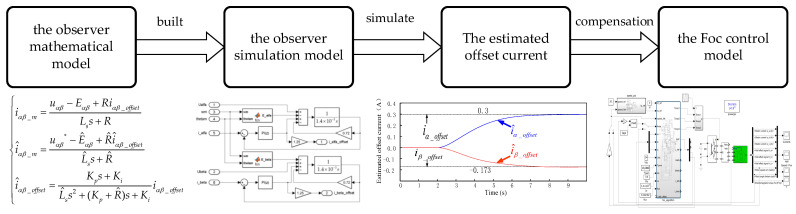
The design process of online detection and compensation scheme for current measurement error based on the PI-type observer.

Under the condition of ensuring that the performance of the observer is satisfied, the observed estimated offset current is used as the error compensation of the offset current to participate in the closed-loop control of the FOC. The design process of online detection and compensation scheme for current measurement error is shown in Figure 7.

### 3.2. Design of the Observer

In the α-β coordinate system, the voltage equation of the surface-mounted PMSM can be presented in the following form [43]:

(9) $$\left\{ \begin{array}{l} u_{\alpha }=Ri_{a}+L_{s}\frac{di_{\alpha }}{dt}-\omega_{e}\Psi_{f}\sin\theta \\ u_{\beta }=Ri_{\beta }+L_{s}\frac{di_{\beta }}{dt}-\omega_{e}\Psi_{f}\cos\theta \end{array}\right.$$

where: *u_α_* and *u_β_* are voltages of α-axis and β-axis, respectively; *i_α_* and *i_β_* are currents of α-axis and β-axis, respectively; *L_s_* is the inductance of the straight and intersection axis, *ω_e_* is the angular velocity of PMSM.

According to Equations (5) and (9), the new voltage equation can be presented as:

(10) $$\left\{ \begin{array}{l} u_{\alpha }=Ri_{\alpha \_m}+L_{s}\frac{di_{\alpha \_m}}{dt}-\omega_{e}\Psi_{f}\sin\theta -Ri_{\alpha \_offset}-L_{s}\frac{di_{\alpha \_offset}}{dt}\\ u_{\beta }=Ri_{\beta \_m}+L_{s}\frac{di_{\beta \_m}}{dt}-\omega_{e}\Psi_{f}\cos\theta -Ri_{\beta \_offset}-L_{s}\frac{di_{\beta \_offset}}{dt} \end{array}\right.$$

In the stable situation, the current offset error can be defined as the disturbance, it behaves as the direct current (DC) error in the α-β coordinate system. The effect of the time derivative on the current is neglected. If *E_α_* = *w_e_ Ψ_f_* sin*θ* and *E_β_* = *w_e_ Ψ_f_* cos*θ*, the disturbance expression and voltage equation (See Equation (10)) can be simplified as:

(11) $$\left\{ \begin{array}{l} d_{\alpha }=Ri_{\alpha \_offset}+L_{s}\frac{di_{\alpha \_offset}}{dt}\approx Ri_{\alpha \_offset}\\ d_{\beta }=Ri_{\beta \_offset}+L_{s}\frac{di_{\beta \_offset}}{dt}\approx Ri_{\beta \_offset}\\ u_{\alpha }=Ri_{\alpha \_m}+L_{s}\frac{di_{\alpha \_m}}{dt}-E_{\alpha }-d_{\alpha }\\ u_{\beta }=Ri_{\beta \_m}+L_{s}\frac{di_{\beta \_m}}{dt}-E_{\beta }-d_{\beta } \end{array}\right.$$

**Figure 8:**
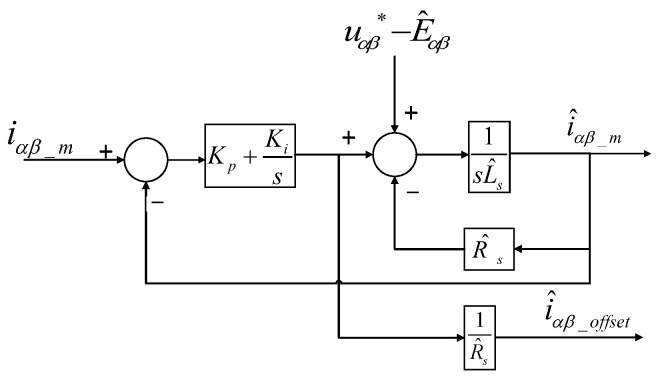
Block diagram of PI-type observer.

Based on the above analysis, a PI-type observer is proposed to estimate the offset current. The block diagram of the PI-type observer is shown in Figure 8; *u*_αβ_^*^ is the command voltage of the PMSM. The input is the difference between the estimated offset current and the measured current in the α-β coordinate system, and the output is the estimated offset current output by the PI-type observer.

If the effect of motor parameters is not considered, and the error of the applied voltage and the estimated back electromotive force is negligible, then the measured current expression, estimated current expression, and the simplified estimated offset current error expression can be presented in the following form:

(12) $$\left\{ \begin{array}{l} i_{\alpha \beta \_m}=\frac{u_{\alpha \beta }\;-\;E_{\alpha \beta }\;+\;Ri_{\alpha \beta \_offset}}{L_{s}s\;+\;R}\\ {\hat{i}}_{\alpha \beta \_m}=\frac{{u_{\alpha \beta }}^{*}\;-\;{\hat{E}}_{\alpha \beta }\;+\;{\hat{R}}_{s}{\hat{i}}_{\alpha \beta \_offset}}{{\hat{L}}_{s}s\;+\;{\hat{R}}_{s}}\\ {\hat{i}}_{\alpha \beta \_offset}=\frac{K_{p}s\;+\;K_{i}}{{\hat{L}}_{s}s^{2}\;+\;(K_{p}\;+\;{\hat{R}}_{s})s\;+\;K_{i}}i_{\alpha \beta \_offset} \end{array}\right.$$

The observer is the PI-type observer with adjustable observer gain. Based on the pole-zero cancellation consideration, if $K_{p}={\hat{L}}_{s}\omega_{c}$, $K_{i}={\hat{R}}_{s}\omega_{c}$, the expressions of *î_αβ_offset_* and *i_αβ_offset_* are simplified to a first-order LPF, and the expression of the simplified estimated offset current can be expressed as:

(13) $${\hat{i}}_{\alpha \beta \_offset}=\frac{\omega_{c}}{s+\omega_{c}}i_{\alpha \beta \_offset}$$

where: *ω_c_* depends on the bandwidth of the first-order LPF.

**Figure 9:**
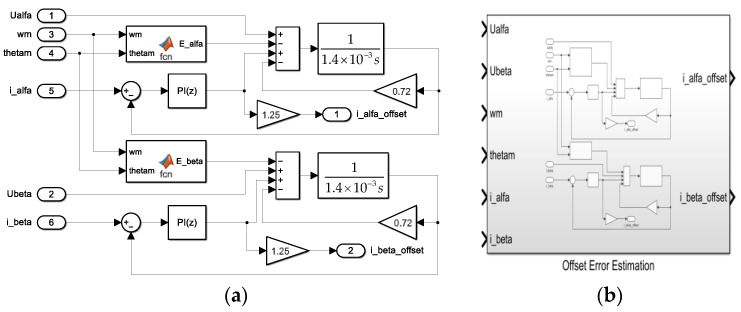
(**a**) The simulation model of PI-type observer in Matlab/Simulink; (**b**) The seal inside of the simulation model.

As shown in Equation (13), *î_αβ_offset_* and *i_αβ_offset_* are equal in the stable case. The simulation model is built in Matlab/Simulink based on the block diagram of the PI-type observer shown in Figure 8. The simulation model and seal inside of it are shown in Figure 9. The model takes phase voltage, phase current, motor angle, and motor speed as inputs, and the output is the estimated offset current of phase current. According to Figure 3 and the simplification process of the third equation in Equation (13), which shows that $\omega_{c}=20$, $R_{s}=0.6$, $L_{s}=1.4\times 10^{-3}$, $K_{p}={\hat{L}}_{s}\omega_{c}$ and $K_{i}={\hat{R}}_{s}\omega_{c}$. In order to reduce the design difficulty, we control the value of ${\hat{L}}_{s}$ constant and adjust the value of ${\hat{R}}_{s}$, we set the value of ${\hat{R}}_{s}$ to 0.72. Because the error in ${\hat{L}}_{s}$ is neglected, in order to obtain the correct value of the current offset, we make a slight adjustment to $\frac{1}{{\hat{R}}_{s}}$ = 1.25. Therefore, PI parameters of the PI controller are $K_{p}=0.0028$ and $K_{i}=14.4$.

**Figure 10:**
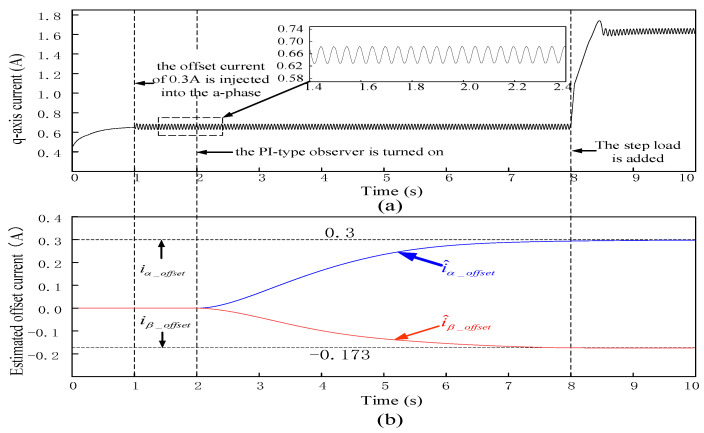
Performance validation of the PI-type observer under no load or step load conditions. During the operation of the PMSM, the offset current of 0.3 A is injected into the a-phase at 1 s, the PI-type observer is turned on at 2 s, and the step load is added at 8 s. (**a**) The waveform of q-axis current of PMSM; (**b**) Estimated value of offset current (blue line and red link are estimated values of offset current in α-axis and β-axis, respectively; 0.3 and −0.173 are theoretical values of offset current in α-axis and β-axis, respectively).

The PI-type observer’s performance is simulated and verified in Matlab/Simulink. In the stable operation stage of the PMSM, the offset current of 0.3 A is injected into the a-phase and b-phase, respectively, to simulate the current offset error caused by the parasitic inductance of sampling resistor. After the offset current of 0.3 A is injected, the output of the PI-type observer and its performance under step torque are explored. The simulation results are shown in Figure 10.

The simulation results show that offset current of 0.3 A is injected into a-phase and b-phase at 1 s. According to Equation (5), those injections will generate current offset errors of 0.3 A and −0.173 A in α-axis and β-axis, respectively. As shown in the Figure 10, the PI-type observer is started to estimate the DC offset error at 2 s of PMSM operation. It can be seen from the Figure 10 that the estimated offset error is consistent with the DC offset error caused by the injected offset current. At 8 s, when the step load is added to the PMSM drive control system, the q-axis current will change abruptly, and the current stabilizes after approximately 0.5 s, but the DC offset error estimated by the PI-type observer is not affected by the step load.

### 3.3. Design of Feed-Forward Compensation Method

**Figure 11:**
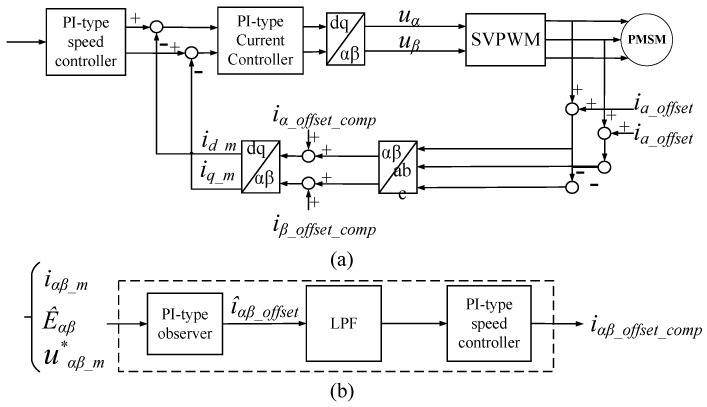
(**a**) The block diagram of DC offset error compensation scheme based on PI-type observer; (**b**) The block diagram of the method for obtaining the current offset error.

In the PMSM drive control system, the DC offset error of phase current output by the PI-type observer is required to be involved in the closed-loop control system of FOC. Based on the above analysis, the proposed DC offset error compensation scheme based on the PI-type observer is shown in Figure 11. 

Where *i_αβ_offset_comp_* is the DC offset error compensation current in α-axis and β-axis.

The complete process of phase current compensation can be described as follows. Firstly, after the DC offset error of the phase current passes through the PI-type observer, the estimated phase current offset error can be obtained. Secondly, the estimated current offset error undergoes processing through the LPF followed by the integrator. The LPF serves to filter the AC component that may be contained in the current offset error. Meanwhile, the integrator’s effect is to ensure that the steady state error of the phase current offset error is 0. Without the integrator, the estimated of the phase current offset error might exhibit steady state error. Eventually, the current offset error compensation of each phase is reinjected into current of α-β axis for error compensation.

### 3.4. Simulation 

**Figure 12:**
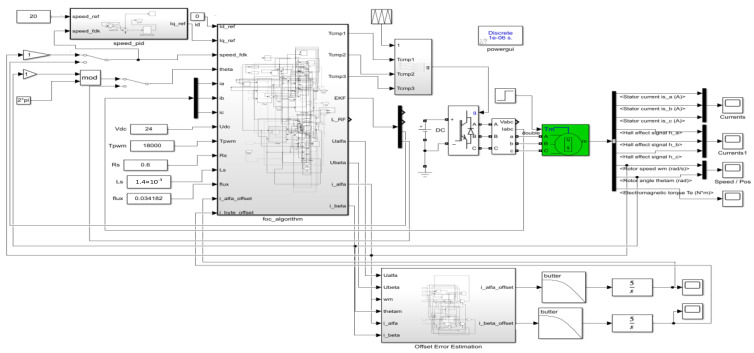
PMSM drive control model for current offset error compensation scheme based on the PI-type observer in Simulink.

The PMSM drive control model for current offset error compensation scheme based on the PI-type observer is build in Matlab/Simulink; the simulation model is shown in Figure 12. The current sampling method in this paper is two-phase current sampling; it needs two integrators to ensure that the steady-state error of the phase current offset error is 0. The simulation sampling time is 1 × 10^−6^ s. The motor model is the surface-mounted PMSM, and the model comes from the model library in Matlab/Simulink. The initial load of PMSM is 0 N·m, and the frequency of the PMSM’s rotation speed is 20 Hz. According to Figure 1, it is known that the current sampling method used in this paper is two-phase current sampling, which needs to capture the phase currents of phase a and phase b separately. According to Equation (5), the current offset errors will produce corresponding current offset errors in α-axis and β-axis, the PI-type observer needs to compensate current offset error of α-axis and β-axis, respectively. Therefore, the model will use two integrators to ensure that the steady state errors of current offset errors are 0.

**Figure 13:**
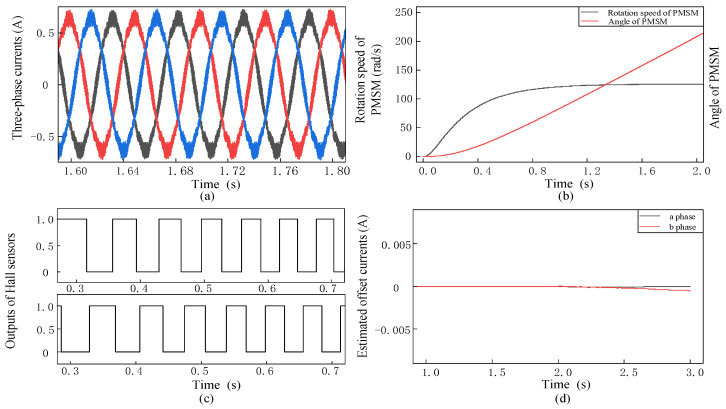
Outputs of each module of the PMSM under the operation of PMSM. The current offset error is 0, and PI-type observer is turned off. (**a**) Waveforms of three-phase currents (black, blue and red lines are phase currents of a, b and c phase, respectively); (**b**) Rotation speed and angle of PMSM (grey and red line are rotation speed and angle of PMSM, respectively); (**c**) Outputs of Hall sensors; (**d**) Estimated values of offset currents (grey and red line are estimated value of offset current in a -phase and b-phase, respectively).

To verify the effect of the PI-type observer on the operation of the PMSM during the normal operation of PMSM, when the current offset errors of a-phase and b-phase are 0. The resulting waveforms of each module are shown in Figure 13.

When the current offset errors are 0, and the PI-type observer participates in the PMSM drive control. As shown in Figure 13, the amplitude of the three-phase current waveform of the PMSM are same, and the phase difference is 120°. As time goes by, the rotation speed is closed to the reference rotation speed, and the angle information is normal. The estimated offset current of the output of the observer is close to 0. The simulation results show that the PI-type observer will not affect the normal operation of PMSM.

**Figure 14:**
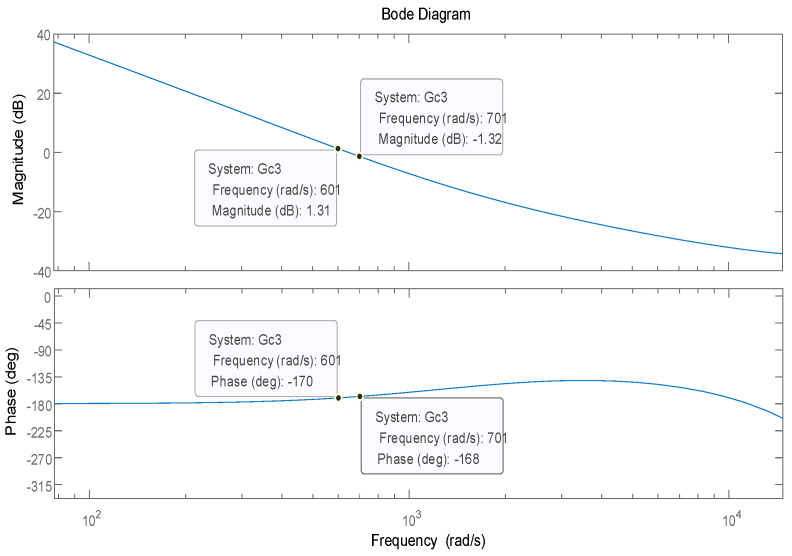
The stability of the PMSM control without the PI-type observer.

**Figure 15:**
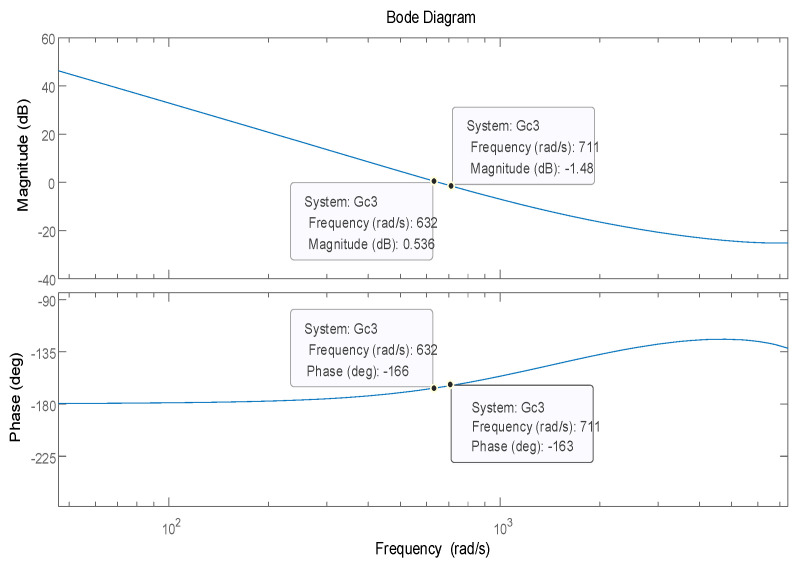
The stability of the PMSM control with the PI-type observer.

In order to verify the effect of the PI-type observer on the stability of the PMSM control system, the stability simulation is carried out in Matlab/Simulink for the PMSM control system without PI-type observer and with PI-type observer, respectively. The stability of the PMSM control without PI-type observer is shown in Figure 14, and the stability of the PMSM control with the PI-type observer is shown in Figure 15.

According to Figure 14 and Figure 15, it can be seen that the presence of the PI-type observer does not affect the stability of the PMSM control system, and can improve the phase margin of the PMSM control system, but the improvement of the phase margin is small.

**Figure 16:**
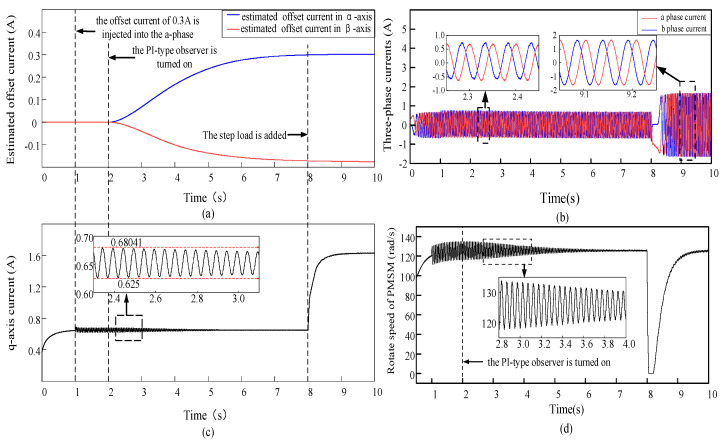
Outputs of each module of the PMSM, when offset currents of 0.3 A and −0.3 A are injected into a-phase and b-phase, respectively. The PI-type observer is turned on. (**a**) Estimated current offset errors, which are output of PI-type observer; (**b**) Three-phase current waveform of PMSM (red line and blue line are a-phase current and b-phase current, respectively); (**c**) The q-axis current waveform of PMSM; (**d**) the rotation speed waveform of PMSM.

To verify the phase current error compensation performance of the PI-type observer with the step load. Offset currents of 0.3 A and −0.3 A are injected into a-phase and b-phase at 1 s during the operation of PMSM, respectively; those are performed to simulate the current sampling error caused by the parasitic inductance of sampling resistor. To observe the three-phase current waveform of the PMSM under the impact of the current offset error. After 2 s of PMSM operation, the PI-type observer is turned on and the phase current error is compensated by the estimated offset current. The step load is added at 8 s to observe the three-phase current information and the operation of PMSM. The output waveforms of each module in the PMSM drive control system are shown in Figure 16. The q-axis current waveform and estimated offset current waveform output by the PI-type observer are shown in Figure 16a,c. The phase current waveforms during the PMSM operation are shown in Figure 16b, and the rotation speed waveform during the PMSM operation is shown in Figure 16d.

As shown in Figure 16a,c, when the offset current is not injected, the q-axis current gradually responds to the current value required at present, and the estimated offset current of the PI-type observer output is approximately equal to 0. At 1 s, the q-axis current oscillates immediately with the offset current injection, and the oscillation amplitude gradually decreases with the error compensation of the offset current. When the step load is applied to the running PMSM drive system at 8 s, it can be found that the PI-type observer is almost unaffected by the step signal and can still output normally. Simulation results demonstrate that the PI-type observer can perform well even under load condition and can estimate the offset current error.

Figure 16b illustrates that the injection of offset currents into a-phase and b-phase at 1 s will induce an abrupt change in the PMSM’s phase currents, and amplitudes of phase currents are not equal. The PI-type observer begins to compensate for current offset errors after 2 s. Over time, the amplitudes of the PMSM phase currents are close to amplitudes before the injection of offset currents. At 8 s, the added step load causes an abrupt change in phase currents, but after about 0.5 s, the stable phase current amplitudes are same, and the angle difference is 120°. The simulation results show that the PI-type observer can also be well involved in the PMSM drive control system with the added step load.

Figure 16d shows that the PMSM experiences a sharp increase in speed upon nor-mal start-up. However, the rotation speed will oscillate due to the injection of offset current at 1 s. At 2 s, the PI-type observer is turned on to compensate for the current offset error, so the rotation speed of PMSM gradually tends to be stable with the error compensation. The step load is added at 8 s, which induces an abrupt change in rotation speed, but the rotation speed gradually stabilizes and ultimately reaches the reference rotation speed and without any oscillations. Simulation results show that the PI-type observer can continuously compensate for the current offset error, and current offset error gradually decreases over time, and the fluctuation of the rotation speed also decreases until it returns to normal speed.

## 4. Experimental Verifications

### 4.1. Construction of the Experimental Platform

**Figure 17:**
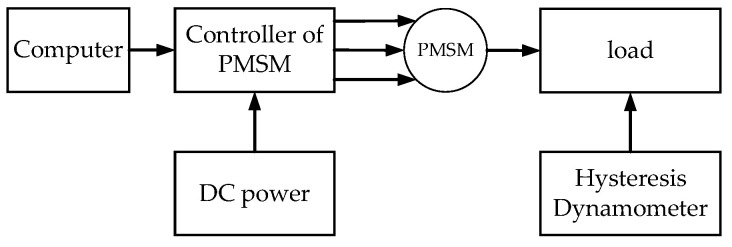
The experimental platform structure diagram of online phase current offset error compensation scheme.

**Figure 18:**
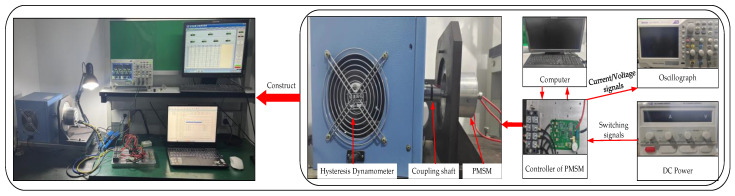
Experimental platform and its structure of online phase current offset error compensation scheme.

To further verify the feasibility of the current offset error compensation based on the PI-type observer proposed in this paper, a PMSM drive control system based on STSPIN32F0A chip is built. The machine parameters are shown in Table 1. The structure of the online current offset error compensation experimental platform is shown in Figure 17, which mainly includes the following: PMSM, PMSM control board (sampling resistors integrated in it), switching power supply, computer, oscillograph. The experimental platform and its structure of online phase current offset error compensation scheme are shown in Figure 18. 

In Figure 18, the PMSM is the driving motor of the automotive electronic water pump, the PMSM is installed on the hysteresis dynamometer, and the PMSM and the hysteresis dynamometer is connected by a coupling shaft. During the PMSM operation, the hysteresis dynamometer imposes the step load to the PMSM. The power supply is the switching power supply with maximum rated current of 30 A.

### 4.2. Results of the Experiment

Before the experiment of the online current offset error compensation scheme, it is essential to verify the effectiveness of the PI-type observer in the open-loop. After downloading the written code to the main control chip, the SEGGER J-SCOPE (Version: V7.90) software is utilized to observe the output waveform of the PI-type observer, and the PI values of the PI-type observer are adjusted so that the estimated α-axis current in the observer is close to the actual measured α-axis current, and the difference is close to 0.

**Figure 19:**
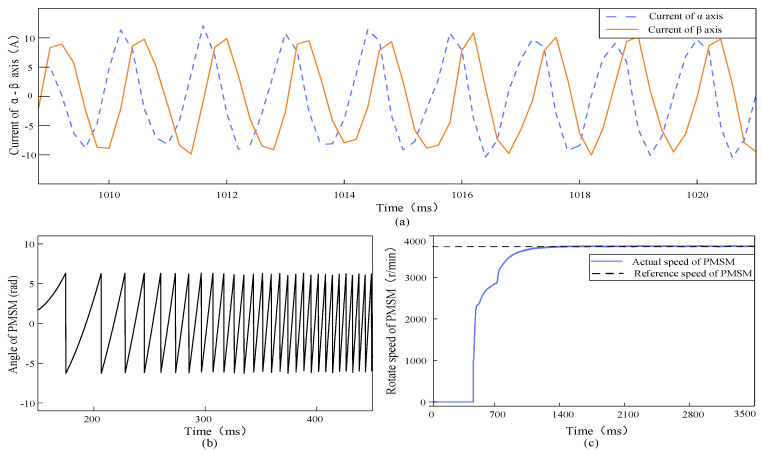
Outputs of each module of the PMSM under the operation of PMSM. The current offset error is 0, and PI-type observer is turned off. (**a**) Current waveforms of PMSM in α-axis and β-axis; (**b**) The angle waveform of PMSM; (**c**) The rotate speed waveform of PMSM.

After downloading the code to the control chip, the reference speed of PMSM is set to 3870 r/Min, and the hysteresis dynamometer is turned on to apply the step load to the PMSM. The current waveforms of PMSM in the α-axis and β-axis are shown in Figure 19a. The angle waveform of PMSM is shown in Figure 19b. The rotate speed waveform of PMSM is shown in Figure 19c.

Figure 19a shows that the current waveforms of the PMSM in the α-axis and β-axis generally show a two-phase symmetrical distribution. Figure 19b shows that the frequency of change of the angle of the PMSM gradually accelerated as the rotational speed of the PMSM increases, and the feedback value of the angle fluctuates between 0 and 2*π*. Figure 19c shows that the PMSM reaches the reference rotational speed at 1 s. Based on the above information, it can be seen that the existence of the PI-type observer does not affect the normal operation of the PMSM.

**Figure 20:**
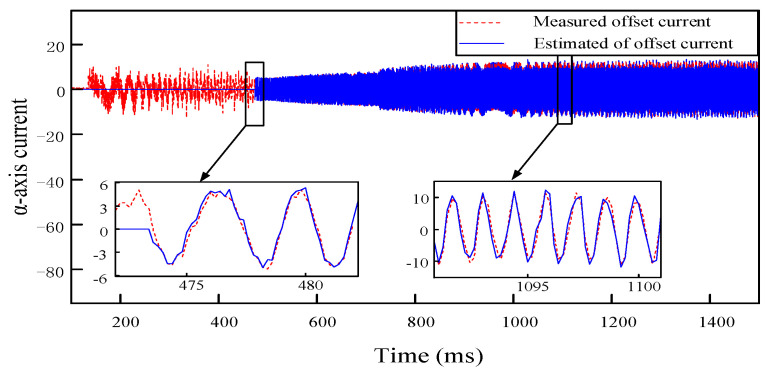
PMSM runs in the stable state, the estimated α-axis current waveform output by the PI-type observer (the blue line) and measured α-axis current waveform of PMSM (the red dotted line). The offset current is 0, and the PI-type observer is turned on.

After ensuring that the difference between the measured current and the estimated current of the PI-type observer is close to 0, the estimated α-axis current waveform of the PI-type observer output and the actual measured α-axis current waveform in the stable operation stage of PMSM are shown in Figure 20.

It can be seen from Figure 20, during the start-up phase of the PMSM, the PI-type observer is turned off, and the estimated current value of α-axis is 0. At 472 ms, the PI-type observer is turned on, and the PMSM is in the high-speed operation state, but the PI-type observer is still able to accurately output the estimated α-axis current. With the PMSM tending to be stable, the estimated α-axis current of the PI-type observer and the actual measured α-axis current are basically the same.

**Figure 21:**
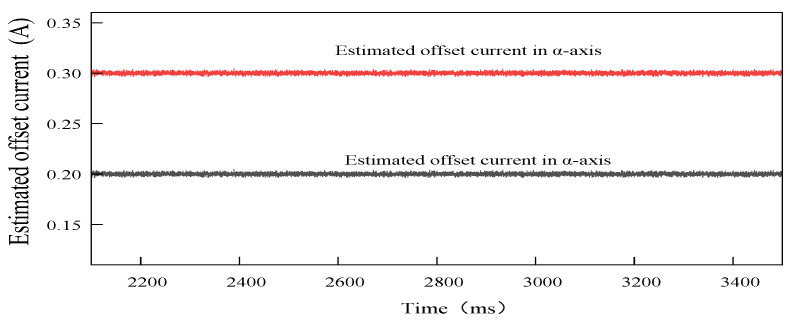
The estimated α-axis offset current waveforms outputs by the PI-type observer in open loop situation. Offset currents of 0.3 A and 0.2 A are injected into a-phase, respectively.

When the offset currents of 0.3 A and 0.2 A are injected into the a-phase of the PMSM, respectively, the estimated offset current waveforms output by the PI-type observer after stabilization are shown in Figure 21. The estimated offset current output by the PI-type observer fluctuates near the actual offset current, and the amplitude of the fluctuation is very small.

**Figure 22:**
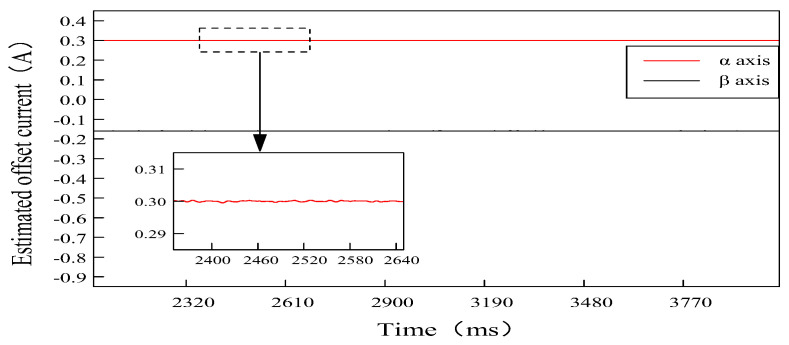
Estimated offset current waveforms of α-β axis, which are outputs by the PI-type Observer in closed loop situation. Offset currents of 0.3 A and −0.3 A are injected into a-phase and b-phase, respectively.

To ensure that the PI-type observer does not impact the normal operation of the PMSM in the open-loop situation, and the compensation performance of the current offset error in the closed-loop situation is further verified. According to the offset error compensation scheme based on PI-type observer in Figure 11, which shows that the estimated offset current by the PI-type observer will finally participate in the closed-loop control system of PMSM as error compensation. Offset currents of 0.3 A and −0.3 A are injected into a-phase and b-phase, respectively; according to the coordinate transformation, it can be seen that offset currents of 0.3 A and −0.173 A will be generated in the α-axis and β-axis, respectively. The estimated offset current waveforms of the PI-type observer in closed-loop situation are shown in Figure 22.

From Figure 22, it can be seen that the PI-type observer has good observation performance, the estimated offset current output by it is basically the same as the actual offset current, and fluctuation range of offset current in α-axis and β-axis are extremely small, which verifies output performance of the PI-type observer in the closed-loop compensation.

Compared with the simulated work, the experimental work further validates the feasibility of the proposed online observation and compensation of current offset error. The comparison results are shown in Table 2.

According to Table 2, the PI-type observer stabilizes the output current offset error and does not affect the normal operation of the PMSM control system in simulations and experiments.

In order to verify the differences between different current offset error compensation methods, this paper also needs to be compared with previous work. Comparison of different current offset error compensation methods is shown in Table 3. 

According to the simulation results and experimental results, this paper aims at the problems of the PMSM output torque pulsation caused by the parasitic parameter of sampling resistor, we propose an online observation and compensation method of current offset error based on PI-type observer, which effectively compensates the current sampling error caused by parasitic inductance of the sampling resistance, and suppresses the speed and torque pulsation of PMSM.

## 5. Conclusions

In this paper, considering the influence mechanism of sampling resistor parasitic inductance and parasitic capacitance on current sampling, the effect of current sampling errors on the torque of PMSM is analyzed, and a mathematical model is established. In order to suppress the torque pulsation of PMSM caused by the current sampling error due to the parasitic inductance of the sampling resistor, an online observation and compensation method of current sampling error based on the PI-type observer is proposed, which effectively suppresses the torque pulsation of PMSM. In the simulations and experiments, an offset current of 0.3 A is injected in a-phase and b-phase, respectively. The PI-type observer can output offset currents of 0.3 A and −0.173 A for compensating the current offset errors in the a-axis and b-axis, respectively, and the presence and operation of the PI-type observer will not affect the normal operation of the PMSM. The simulation and experimental results demonstrate the following.

- The sampling resistor in PMSM applications has a parasitic inductive-capacitive nature that causes current sampling bias, which further causes torque pulsation and leads to degradation of motor performance.
- The paper proposes a PI-type observer that can effectively obtain the offset error estimate value of phase currents by the online observation method.
- The paper proposes an online error compensation method that can rapidly compensate for phase current offset errors, effectively reducing motor rotation speed and torque pulsation caused by such errors, while exhibiting good dynamic performance.

Despite the limitations in the analysis, the proposed measurement method of current offset error and the compensation scheme of current offset error show the value of the new method, and the feasibility of the method is verified by simulations and experiments. Future studies and more in-depth validation should include the following:

- For the impact of the parasitic inductance of the sampling resistor on the PMSM drive control system, it is known that the offset current caused by it is periodic, this periodic signal can be further studied to eliminate the impact of the parasitic inductance of sampling resistor on the PMSM drive control system.
- When PMSM is running at low speed, operating frequency of the motor and the sampling resistor parasitic capacitance are small, many scholars ignore the impact of the sampling resistor parasitic capacitance on the PMSM drive control system. However, when the PMSM is running at high speed, the impact of the sampling resistor parasitic capacitance on the PMSM drive control system needs to be further clarified.

## Figures and Tables

**Table 1:** The machine parameters of PMSM.

Symbol	Parameter	Value
$P_{n}$	Rated power	420 W
	Rated current	15 A
p	Pole pairs	2
$R_{s}$	Stator resistance	9 mΩ
$L_{d}$	d-axis inductance	93 μH
$L_{q}$	q-axis inductance	93 μH
$\Psi_{f}$	Flue	0.0175 *W_b_*
$V_{in}$	Input voltage of power stage	12 V

**Table 2:** Comparison of experimental results and simulated results.

Type of Work	Rotation Speed	Injected Offset Current	Response Time	Outputs of PI Observer	Operation of PMSM
simulation	120 rad/s	0.3 A ^1^	5 s	0.3 A ^3^	normal
		−0.3 A ^2^		−0.173 A ^4^	
experiment	63 rad/s	0.3 A ^1^	622 ms	0.3 A ^3^	normal
		−0.3 A ^2^		−0.175 A ^4^	

^1^ The offset current was injected into a-phase. ^2^ The offset current was injected into b-phase. ^3^ The estimated offset current of α-axis. ^4^ The estimated offset current of β-axis.

**Table 3:** Comparison of different current offset error compensation methods in five previously published works.

Works	Rotation Speed	Method of Compensation	Compensating Object	Difficulty	Scope of Application
Ref. [24]	167 rad/min	Online	Current of q-axis and d-axis	Moderate	Low speed
Ref. [26]	500 rad/min	Online	Current of q-axis and d-axis	Easy	Low speed
Ref. [27]	150 rad/min	Online	Current of q-axis and d-axis	Easy	Low speed
Ref. [28]	1000 rad/min	Offline	Current of a-axis and b-axis	Difficult	Low speed
Ref. [29]	2000 rad/min	Online	Current of q-axis and d-axis	Difficult	Middle speed
Ours works	3780 rad/min	Online	Current of α-axis and β-axis	Easy	Middle speed

## Data Availability

The data presented in this study are available on request from the corresponding author.
